# Determinants of early interruption of exclusive breastfeeding at Dollo Ado refugee camps, Dollo Ado district, Ethiopia

**DOI:** 10.11604/pamj.2023.45.105.35949

**Published:** 2023-06-26

**Authors:** Dawit Getachew, Desta Haftu, Tewodros Yosef

**Affiliations:** 1 Department of Public Health, School of Public Health, College of Medicine and Health Sciences, Mizan-Tepi University, Mizan Teferi, Ethiopia,; 2 School of Public Health, College of Medicine and Health Sciences, Arba Minch University, Arba Minch, Ethiopia

**Keywords:** Exclusive breastfeeding, early interruption, determinants, Dollo Ado refugee camps, Ethiopia

## Abstract

**Introduction:**

nearly three-quarters of infants younger than six months were not exclusively breastfed globally. Despite some research indicating what factors influence early exclusive breastfeeding interruption in Ethiopia's stable population, there is little evidence indicating what factors influence exclusive breastfeeding interruption in vulnerable populations such as refugee camps. Therefore, this study aimed to determine the factors that contributed to the early termination of exclusive breastfeeding in Ethiopian refugee camps in the Dollo Ado district.

**Methods:**

a case-control study was conducted at the Dollo Ado refugee camps from April 05^th^ to 25^th^, 2017. The eligible 112 cases and 224 controls were identified using the 24-hour recall method. The information was gathered using an interviewer-administered questionnaire that was pretested and organized. Logistic regression analysis was computed to assess the effect of independent variables.

**Results:**

the determinants for early interruption of exclusive breastfeeding were not counseled about infant feeding during antenatal care follow-up (adjusted odds ratio (AOR =5.87, 95% CI [2.61-13.1]), not counseled about infant feeding during postnatal care service use (AOR= 4.33, 95% CI [2.71-10.8), breastfeeding problem (AOR= 5.62, 95% CI [4.55-15.2]) and late initiation of breastfeeding (AOR= 4.79, 95% CI [28-10.1]).

**Conclusion:**

in this study, early termination of exclusive breastfeeding was caused by breastfeeding problems and late commencement of breastfeeding, as well as not receiving infant feeding advice during antenatal care or postnatal care. The results of this study highlight the significance of concentrating on newborn and young child feeding counseling during prenatal and postnatal care services in order to promote exclusive breastfeeding. In addition, health providers should educate parents on the significance of starting exclusive breastfeeding on time and obtaining help right away if there is a problem, such as breast soreness or the infant refusing to eat due to oral trash, to avoid early exclusive breastfeeding interruption.

## Introduction

For infants less than six months, exclusive breastfeeding (EBF) is one of the elements of ideal infant and young child feeding (IYCF), which is defined as supplying only human breast milk, including expressed human milk [1]. The World Health Organization (WHO) recommends that infants less than six months be breastfed exclusively since it is a healthy and sufficient feeding method that helps to increase infant survival and protects against infections, chronic diseases, and growth problems [2-6]. Around 70% of children under six months old were not exclusively breastfed in 2014 [7]. In Africa, 68% of infants are interrupted from receiving only breast milk, compared to nearly 50% in Ethiopia [8]. Sixty percent of the 10.9 million children who die each year around the world are victims of malnutrition, which either directly or indirectly results from improper feeding during infancy [9]. In sub-Saharan Africa alone 1.16 million infants die in their first month of life [10], but we could have saved 800,000 infant deaths by practicing EBF [11]. Infection and diarrhea were the two main causes of infant mortality in Africa, and both are easily preventable through immunization and EBF [12,13]. The baby-friendly hospital Initiative was implemented as a component of the WHO's global baby and young child nutrition strategy (IYCF). However, the development was quite marginal [14]. In Ethiopia, just three of the 24 refugee camps used IYCF practices [15,16]. Social services for refugees are not well established [17]. As a result, insufficient health-care services are unavoidable, posing health risks to both mother and child, including the early termination of EBF [16]. As a result, extra emphasis should have been paid to IYCF, particularly safeguarding and supporting breastfeeding, which is vital not only in an emergency, but also has long-term effects on infant health and women's future feeding preferences [18]. Several studies have done previously regarding what causes early interruption of exclusive breastfeeding and include bottle feeding and artificial ingredients in breast milk substitutes [19], mother working outside the home [20-25], breastfeeding problem of the mother like a cracked nipple [20,26], lack of prior breastfeeding experience [20], pre-lacteal feeding [21,26,27], being female child and increasing age of infant [21,22,24,26,28,29] and mothers limited nighttime breastfeeding [20] were the factors that increase the chance of early interruption of EBF. Other studies also revealed that increased age of the mother [26,28], better educational status [26], good income [29], advice on IYCF during antenatal care(ANC) and postnatal care (PNC) services [26,28-30], spontaneous vaginal delivery and delivery at health institution [20,21], early initiation of breastfeeding [24,30,31], knowledge of the mother on IYCF practice [21,30], feeding infants the first breast milk (colostrum) [20,32] were the factors associated with less chance of early interruption of EBF. Ideal infant and young child feeding best practices are less likely to be followed in an emergency than they would be under regular conditions. Despite the fact that there is some data about what causes early EBF interruption in Ethiopia's steady population, however, there isn't a shred of information about what causes EBF disruption in insecure populations such as refugee camps. Any intervention aimed at promoting exclusive breastfeeding may only be used if enough data is available on the subject. Therefore, this study aimed to assess the determinants of early interruption of exclusive breastfeeding at refugee camps of Dollo Ado district in Ethiopia.

## Methods

**Study design, setting and period:** a case-control study was conducted at Dollo Ado refugee camps, Dollo Ado district from April 05^th^ to 25^th^, 2017. Dollo Ado refugee camps are found in Dollo Ado district, Liben Zone, Ethiopia Somali Region at 935km southeast of Addis Ababa, the capital city of Ethiopia. Dollo Ado district refugee camps (Bokolmayo, Melkadida, Kobe, Heliwoyn and Bura amino camps) are the home of 251,987 Somali refugees population. The Ethiopian Government protects refugees in collaboration with national, international NGOs, and United Nations (UN) agencies.

**Sample size determination:** the sample size was calculated using Open Epi version 3 statistical software for unmatched case-control studies considering the following assumption: two-sided 95% confidence level (Zα/2=1.96), power of 80%, the ratio of control to cases 2:1 (r = 2), odds to be detected 1.99 and 42% [33] of the control group to be exposed and adding 10% for non-response rate compensation. The final sample size was 336 (112 cases and 224 controls).

**Sampling technique:** for each sub-camp of the Dollo ado refugee camps, the total sample size was distributed proportionally. During the study period, screening questions using a 24-hour recall were given to all mothers who have infants younger than six months old at refugee camps in the Dollo Ado district and who come for ration collection. A 24-hour recall method, which is advised by the World Health Organization to identify cases and controls, was used to inquire about an infant's nursing status on the day when rations were distributed. Mothers who have an infant less than six months and responded “yes” for the screening question, which was “have you given anything to eat or drink to your infant in the previous 24 hours or the previous day and night?” considered as cases and who answered “no” considered as controls. In the Dollo Ado refugee camps, mothers of index infants who were less than six months old at the time of ration registration were identified and coded as part of the sampling procedure. For every one eligible case and two consecutive mothers in the controls were interviewed in the aforementioned refugee camps. The study included all sampled moms who had a child younger than six months at each of the five locations of the Dollo Ado district refugee camp during the study period. Mothers who were really unwell and unable to react were not included in the study.

### Operational definitions

**Exclusive breastfeeding:** was defined as giving only human breast milk including expressed human milk to infants less than six months in the previous 24 hours, otherwise not.

**Cases:** were those who have interrupted breastfeeding.

**Controls:** were defined as those refugees exclusive breastfeeding at the time of the interview.

**Knowledge of a mother on exclusive breastfeeding:** was defined as the mother´s information on the advantages and recommended duration of exclusive breastfeeding. Those who scored mean value and above considered as good knowledge, otherwise poor.

**Timely initiation of breastfeeding:** infants who put to the breast within one hour of birth. Prelacteal feeding is when infants receive nutrition other than breast milk for the first three days of life before being breastfed.

**Data collection procedure:** mothers who had been identified as cases and controls were face-to-face questioned using a standardized, tested questionnaire that was adopted from Ethiopian Demographic and Health survey (EDHS) 2016 [33]. The questionnaire was written in English, translated into Somali, and then retranslated back into English to ensure consistency. The questionnaire asked about the mothers' sociodemographic characteristics, their use of health services, and their awareness of EBF. Five supervisors (one for each camp) and ten trained data collectors (two for each camp) were hired. Data collectors and supervisors received training on the purpose and methodology of data gathering as well as how to handle questions that are unclear. Before beginning the real data collection, the questionnaire was pre-tested in similar settings (5% of the sample size) to improve the quality of the data.

**Statistical analysis:** the data were analyzed using statistical package for the social sciences (SPSS) version 20. Descriptive statistics were undertaken. The results of the study were expressed in terms of frequencies, percentages and presented using tables. To determine the factors connected to the outcome variable, binary logistic regression analysis was used. To minimize the impact of confounding, independent variables with a p-value 0.2 in the bivariate analysis were fitted into the multivariable analysis. P-values under 0.05 were used to define the level of statistical significance. The Hosmer-Lemeshow goodness-of-fit test revealed that the model adequately fit the data (P = 0.7), indicating that the model was good enough.

**Ethics approval and consent to participate:** the Institutional Review Board at Arba Minch University granted its ethical approval. With the reference number AMUIRB/88/2017, ethical approval was issued on January 25, 2017. The participants in the study were told of its objectives, their right to decline participation, the study's anonymity and confidentiality policies, and the fact that it was carried out in conformity with the Helsinki Declaration. The participants in the study provided their written informed permission.

## Results

**Sociodemographic characteristics:** of the 112 cases and 224 controls recruited, 103 cases and 208 controls were interviewed yielding the response rate of 92% and 93% respectively. All study participants were Muslim by religion and speak the Somali language. The mean (± SD) age of mothers was 25 (± 5) years. Rahan-wayn is the largest ethnic group, followed by Merihan and Hawiye. The majority of the mothers were married, uneducated, and all most all the mothers work inside the home (Table 1). The mean (± SD) age of infants was 3 (± 1) months.

**Table 1:** sociodemographic characteristics of study participants at refugee camps of Dollo Ado district in Ethiopia (N= 311)

Variables	Categories	Cases, n (%)	Controls, n (%)
**Ethnicity**	Rahan-wayn	59 (57)	118 (57)
	Hawiye	25 (24)	36 (17)
	Marehan	18 (17)	44 (21)
	Others*	1 (1)	10 (5)
**Marital status**	Married	84 (82)	167 (80)
	Single	19 (18)	41 (20)
**Educational status**	Educated	6 (6)	25 (12)
	Uneducated	97 (94)	183 (88)
**Occupational status**	Work at home	84 (82)	179 (86)
	Work outside home	19 (18)	29 (14)
**Parity**	Primipara	28 (27)	55 (26)
	Multipara	75 (73)	153 (74)
**Birth interval**	< 2 years	71 (69)	135 (65)
	≥ 2 years	32 (31)	73 (35)

* Bantu, Darod, and Dir

**Health service-related, medical and baby factors:** mothers who had ANC advice on breast-feeding were 56% and 92% among cases and controls respectively. Regarding initiation of EBF 46% of cases and 85%, controls were initiated timely. Postnatal care service utilization of the mother in the study is 73% in cases and 94% in controls; out of this, 57% of cases and 88% of control have PNC advice on IYCF. Among mothers who had breastfeeding problems, 37% and 8% were cases and controls respectively. The proportion of infants with prelacteal feeding was 96% among cases and 87% among controls (Table 2).

**Table 2:** health service-related, medical, and baby factors of interruption of exclusive breastfeeding among study participants at refugee camps of Dollo Ado district in Ethiopia (N= 311)

Variables	Categories	Cases, n (%)	Controls, n (%)
**Antenatal care advice on infant feeding**	Yes	58 (56)	192 (92)
	No	45 (44)	16 (8)
**Postnatal advice on infant feeding**	Yes	59 (57)	183 (88)
	No	44 (43)	25 (12)
**Problem of breastfeeding**	Yes	37 (36)	16 (8)
	No	66 (64)	192 (92)
**Initiation of breastfeeding**	Timely initiation	47 (46)	177 (85)
	Late initiation	56 (54)	31 (15)
**Pre-lactal feeding**	Yes	99 (96)	181 (87)
	No	4 (4)	27 (13)
**Gender of the baby**	Male	56 (54)	108 (52)
	Female	47 (46)	100 (48)
**Baby health status**	Good	25 (24)	42 (20)
	Bad	78 (76)	166 (80)
**Knowledge of mother on exclusive breastfeeding**	Good	62 (60)	164 (79)
	Poor	41 (40)	44 (21)

**Determinant early interruption of exclusive breastfeeding:** the potential related factors were examined using bivariate analysis. Women who received IYCF counseling during ANC and PNC, breast problems, mothers' awareness of exclusive breastfeeding, and late breastfeeding initiation were the factors linked to early interruption of exclusive breastfeeding. After adjusting for confounding variables, not counseled about infant feeding during ANC follow-up (adjusted odds' ratio (AOR =5.87, 95% CI [2.61-13.1])), not counseled about infant feeding during PNC service use (AOR= 4.33, 95% CI [2.71-10.8]), breastfeeding problem (AOR= 5.62, 95% CI[4.55-15.2]) and late initiation of breastfeeding (AOR= 4.79, 95% CI [2.28-10.1]) were the determinants of early interruption of EBF (Table 3).

**Table 3:** determinants for early interruption of exclusive breastfeeding among study participants at refugee camps of Dollo Ado district in Ethiopia (N= 311)

Variables	Categories	Early interruption of exclusive breastfeeding		COR (95% CI)	AOR (95%CI)
		Cases, n (%)	Controls, n (%)		
**Infant and young child feeding advice during antenatal care**	Yes	58 (56)	192 (92)	1	1
	No	45 (44)	16 (8)	9.31 (4.45-14.4)**	5.87 (2.61-13.1)**
**Infant and young child feeding advice during postnatal care**	Yes	59 (57)	183 (88)	1	1
	No	44 (43)	25 (12)	5.46 (4.30-15.2)*	4.33 (2.71-10.8)**
**Problem on breastfeeding**	Yes	37 (36)	16 (8)	6.73 (4.67-16.1)*	5.62 (4.55-15.2)**
	No	66 (64)	192 (92)	1	1
**Knowledge of mother on exclusive breastfeeding**	Good	62 (60)	164 (79)	1	1
	Poor	41 (40)	44 (21)	2.46 (1.48-4.15)*	1.61 (0.77-3.35)
**Initiation of breast feeding**	Timely	47 (46)	177 (85)	1	1
	Late	56 (54)	31 (15)	6.80 (4.16-12.6)**	4.79 (2.28-10.1)**

AOR: adjusted odds ratio, CI: confidence interval, COR: crude odds ratio, *p-value < 0.2, **p-value < 0.05

## Discussion

Social services for refugees are not well established [17]. The inability of mothers to access resources that encourage breastfeeding due to a lack of infrastructure and adequate health care services may result in the early cessation of EBF [16]. Providing special attention to IYCF, particularly safeguarding and sustaining breastfeeding, is critical not just in an emergency, but also in terms of a child's long-term health and a woman's future feeding preferences [18]. Based on the above scenario, we aimed to assess the determinants of early interruption of exclusive breastfeeding at Dollo Ado refugee camps in Dollo Ado district, Ethiopia. Mothers who did not get infant feeding advice during ANC follow-up were 5.9 times more likely to stop exclusive breastfeeding than mothers who did. This finding was supported by studies done in Ethiopia and Nigeria [20,21,28,29]. This could be explained by the fact that the mother who had IYCF counseling on EBF will have time to prepare herself on the appropriate type of infant feeding, which could be due to the awareness created by health professionals especially midwives who teach mothers about appropriate infant and young child feeding practices [34] during ANC follow-up. Mothers who were not advised on infant feeding during PNC service use were 4.3 times more likely than mothers who were counseled on infant feeding during PNC service use to interrupt exclusive breastfeeding. This finding was in line with studies done in Ethiopia [20,24]. Compared to women who did not have breastfeeding problems, mothers who had problems were 5.6 times more likely to interrupt exclusive breastfeeding This finding was in line with a multi-center study conducted in eight different countries [32]. This is may be due to inflammation of the breast, a cracked nipple that causes pain to the mother [35,36]. Mothers who started breastfeeding recently or within the first hour of delivery were 4.8 times more likely to stop than mothers who started breastfeeding shortly after birth or within the first hour of birth. This could be due to infants' ability to adapt to what they've been given just after birth and strengthen their link with their mothers. This may influence infant feeding preferences; for example, a mother who breastfeeds her baby right away may continue to feed her baby solely breast milk because she believes the baby won't be able to adapt to other foods. This conclusion was supported by research conducted in Ethiopia [26,29,30]. The proportion of infants with prelacteal feeding was 96% among cases and 87% among controls. The finding was higher than a study done by Gedefaw M *et al*. [37] revealed that the proportion of pre-lacteal feeding among cases, and controls was 87%, and 17%, respectively. This may result in a high number of infant deaths attributable to inappropriate feeding occurring during the first 6 months of life [38].

**Strengths and limitations:** this study's use of a 24-recall method, which lessens mothers' recall bias, and its attempt to identify the determining elements of EBF interruption on the most susceptible population at most peripheries are both strengths. Its weakness, though, was that it didn't deal with the problem's qualitative component. Due to a lack of literature in the refugee population, the study results were also contrasted with those from a stable population.

**Funding:** this study did not received monetary fund, but the researchers have received transport and stationary support from Ethiopian National Intelligence and Security Service- Administration of Refugee and Returnee Affair and NOGs working in Dollo Ado refugee camps.

## Conclusion

In this study, early termination of exclusive breastfeeding was caused by breastfeeding problems and late commencement of breastfeeding, as well as not receiving infant feeding advice during antenatal care or postnatal care. The results of this study highlight the significance of concentrating on newborn and young child feeding counseling during prenatal and postnatal care services in order to promote exclusive breastfeeding. In addition, health providers should educate parents on the significance of starting exclusive breastfeeding on time and obtaining help right away if there is a problem, such as breast soreness or the infant refusing to eat due to oral trash, to avoid early exclusive breastfeeding interruption.

### What is known about this topic

- *In Africa, 68% of infants are interrupted from receiving only breast milk, compared to nearly 50% in Ethiopia;*
- *In sub-Saharan Africa alone 1.16 million infants die in their first month of life, but we could have saved 800,000 infant deaths by practicing exclusive breastfeeding;*
- *Infant and young child feeding best practices are less likely to be followed in an emergency than they would be under regular conditions*.

### What this study adds

- *The proportion of infants with prelacteal feeding was 96% among cases and 87% among controls;*
- *The determinants for early interruption of exclusive breastfeeding were not counseled about infant feeding during antenatal care follow-up, not counseled about infant feeding during postnatal care service use, breastfeeding problem and late initiation of breastfeeding*.
